# Supplementary material for: The xIV-LDDMM toolkit of image-varifold based technologies for mapping 3D images and spatial-omics across scales
Source: Commun Biol. 2025 Sep 30;8:1401. doi: 10.1038/s42003-025-08800-7 (PMC12484866; doi:10.1038/s42003-025-08800-7)
Supplement: Supplementary file 3 — Description of Additional Supplementary Materials [file 42003_2025_8800_MOESM3_ESM.pdf]

Description of Additional Supplementary Files

File name: Supplementary Data 1

Description: Raw data associated to figures 4m and 4n and supplementary figure 2a.

File name: Supplementary Data 2

Description: Raw data associated to figures 8h and 10c and supplementary figure 3.

File name: Supplementary Data 3

Description: Raw data associated to figure 7y.
